# Dual-targeting class I HDAC inhibitor and ATM activator, SP-1-303, preferentially inhibits estrogen receptor positive breast cancer cell growth

**DOI:** 10.1371/journal.pone.0306168

**Published:** 2024-07-15

**Authors:** Mira Jung, Nicole Nicholas, Scott Grindrod, Anatoly Dritschilo

**Affiliations:** 1 Department of Radiation Medicine, Georgetown University School of Medicine, Washington, DC, United States of America; 2 Department of Biochemistry & Molecular & Cellular Biology, Georgetown University School of Medicine, Washington, DC, United States of America; 3 Shuttle Pharmaceuticals, Inc., Rockville, Maryland, United States of America; University of East Anglia, UNITED KINGDOM OF GREAT BRITAIN AND NORTHERN IRELAND

## Abstract

Dual-targeting chromatin regulation and DNA damage repair signaling presents a promising avenue for cancer therapy. Applying rational drug design, we synthesized a potent dual-targeting small molecule, SP-1-303. Here, we report SP-1-303 as a class I isoform selective histone deacetylase (HDAC) inhibitor and an activator of the ataxia-telangiectasia mutated protein (ATM). *In vitro* enzymatic assays demonstrated selective inhibition of HDAC1 and HDAC3. Cellular growth inhibition studies show that SP-1-303 differentially inhibits growth of estrogen receptor positive breast cancer (ER+ BC) cells with effective growth inhibition concentrations (EC_50_) for MCF-7 and T47D cells ranging from 0.32 to 0.34 μM, compared to 1.2–2.5 μM for triple negative breast cancer cells, and ~12 μM for normal breast epithelial cells. Western analysis reveals that SP-1-303 decreases estrogen receptor alpha (ER-α) expression and increases p53 protein expression, while inducing the phosphorylation of ATM and its substrates, BRCA1 and p53, in a time-dependent manner in ER+ BC cells. Pharmacokinetic evaluation demonstrates an area under the curve (AUC) of 5227.55 ng/ml × h with an elimination half-life of 1.26 h following intravenous administration in a rat model. Collectively, SP-1-303 emerges as a novel second generation class I (HDAC1 and HDAC3) selective HDAC inhibitor, and ATM activator, capable of modulating ER expression, and inhibiting growth of ER+ BC cells. Combined targeting of class I HDACs and ATM by SP-1-303 offers a promising therapeutic approach for treating ER+ breast cancers and supports further preclinical evaluation.

## Introduction

Breast cancer is the most common cancer in women. Risk factors include mutations in tumor suppressor genes as well as environmental exposures. Based on U.S. Breast Cancer Statistics, about 12% of women will develop invasive breast cancer over the course of their lifetime [1]. In 2023, 297,790 estimated new cases of breast cancer were expected to lead to 43,170 death in the U.S. [2]. There is an urgent need for innovative breast cancer therapeutics.

Patients presenting with breast cancer may receive treatment, with surgery, radiation therapy, hormonal therapy, chemotherapy, or immune therapy, based on stage and extent of disease. The treatment options are determined by levels of hormone receptors (ER/PR+/-), epidermal growth factor type 2 receptors (HER2/neu+/-), or the lack of these receptors [triple negative cancers (TNC with ER/PR/HER2 negative)] in the tumor tissue. The level of estrogen or progesterone receptors (ER+/PR+) in cancer tissues also correlates with risk [3]. In the United States, 70% of breast cancers are initially ER positive and respond to tamoxifen or aromatase inhibitors [1]. Upon disease progression, patients with cancers with BRCA gene mutations are candidates for treatment with PARP inhibitors [4]. Other patients receive chemotherapies that act on rapidly dividing cancerous and normal cells. There is a need for targeted-therapies to selectively treat cancers and reduce toxicities and side effects.

Epigenetic silencing of tumor suppressors involved in DNA damage repair (DDR) pathways is a hallmark of tumorigenesis. Perturbation of chromatin regulation and DDR pathways are important risk factors of cancer progression, underlying interventions to alter chromatin regulator activities and to enhance DDR signaling as a new direction for cancer treatment [5,6]. Four pan-HDAC inhibitors [Vorinostat (SAHA), Belinostat (PXD-101), Romidepsin (FK-228) and Panobinostat (LBH589)] have received FDA approvals, primarily for the treatment of hematological malignancies. However, success in the treatment of solid tumors using HDAC inhibitors has been limited and is associated with off-target effects and toxicities [7–9]. Currently, Panobinostat’s FDA approval for treatment of refractory multiple myeloma and Romidepsin for the peripheral T-cell lymphoma indications have been withdrawn from the US market due to lack of feasibility for clinical applications [10,11].

Recent studies have shown that overexpression of HDACs in many cancer types is linked to increases in cell proliferation and resistance to genotoxic-based therapies and that class I HDACs are associated with poor prognosis [12,13]. Moreover, pharmacological inhibitors of class I HDACs have been shown to enhance tumor response to therapies by inducing epithelial differentiation [14–16]. The benzamide derivative entinostat was reported as a class I inhibitor (HDAC1 and HDAC3 with IC_50_ of 0.51 μM and 1.7 μM, respectively) that demonstrated preclinical and Phase II clinical efficacy in ER+ BC with an unclear mechanism of action [17,18]. The combination of exemestane and entinostat did not improve survival in aromatase inhibitor resistant hormone receptor (HR)-positive, HER2-negative breast cancers [19], demonstrating the limitation of this drug combination approach.

Perturbation of chromatin regulation and DNA damage repair (DDR) pathways are important risk factors in tumor development. The tumor suppressor gene, BRCA1, a substrate of the ataxia-telangiectasia mutated (ATM) gene product, is part of a complex of molecules in the repair of DNA double-strand breaks (DSBs), and loss of BRCA1 contributes to progression of both, sporadic and inherited breast cancers [20]. Furthermore, *ATM* is a frequently aberrant gene with loss of heterozygosity in ~40% of human sporadic BC [21]. In this study, we observed that our rationally designed drug candidate, SP-1-303, offers a dual action by preferentially inhibiting the class I HDAC1 and HDAC3, and enhancing ATM activation. Cellular studies demonstrated that the compound inhibits ER^+^ BC cell growth (4-8-fold) as compared to the effects on ER^-^ TNBC cell growth, supporting evaluation for a possible role in treating ER^+^ breast cancers. Furthermore, SP-1-303 arrested ER^+^ breast cancer cells in the G1 phase of the cell cycle, a radiation sensitive phase, suggesting that SP-1-303 may offer a synergistic effect in combination with radiation.

## Methods & materials

### Cell culture and materials

MCF7 and T-47D cells were cultured in RPMI 1640 Medium supplemented with L-glutamine, 10% FBS and 1% Streptomycin. MCF10A were cultured in DMEM supplemented with 5% CCS. MDA-MB-231 cells were cultured in L-15 medium, 2 mM glutamine, and 15% FBS. HCC 1937 were cultured in McCoy’s 5A medium with 2mM L-glutamine and 10% FBS. Antibodies were purchased from Abcam, Santa Cruz, and additional compounds, including tamoxifen and SAHA, were obtained from Selleckchem.

### *In vitro* HDAC assay

*In vitro* HDAC enzyme inhibition assays utilized HeLa cell extract for pan-HDACs with the substrate Fluor-Lys following the manufacturer’s protocol (BIOMOL/ENZO, Plymouth Meeting, PA). For isoform assays, purified HDAC1, HDAC3, and HDAC6 recombinant proteins produced by expressing epitope-tagged full-length proteins in the sf9 baculovirus expression vector system were purchased from PBS Bioscience (San Diego, CA). Inhibitor activity was assessed using an HDAC Fluorimetric Assay/Drug Discovery Kit (cat# 5004). Briefly, reactions were set up in 96-well plates with a total volume of 50 μL HDAC assay buffer (50 mM Tris-HCl, pH 8.0, 137 mM NaCl, 2.7 mM KCl, 1 mM MgCl2) containing HDAC1, HDAC3, or HDAC6, testing compounds, and substrates. SAHA served as the positive control, while the negative control employed 0.05% DMSO as the vehicle. The reaction commenced with the addition of HDAC substrate at room temperature and proceeded for 30 minutes. Termination of the reaction was achieved by adding 50 μL of Fluor de Lys (TM) Assay Developer. The fluorophore was excited at 360 nm and emitted light at 460 nm. Relative fluorescence was measured using a FlexStation II plate reader (Molecular Devices, Sunnyvale, CA). Raw data (relative fluorescence units) were plotted against the molar concentration of test compounds (logarithmically) and fitted to a parameter logistic function to calculate IC_50_ using Prism 10.2.2 (GraphPad Software, La Jolla, CA).

### Cytotoxicity assay

Cells were seeded for 24 hours in a 96 well plate in doublets, followed by a 48-hour drug treatment with the drug compounds [SP-1-303, SAHA (Vorinostat) and Tamoxifen] at concentration ranges of 0.25–50 ∝M. To determine the absorbance, a colorimetric assay Cell Counting Kit-8 (CCK8) (Millipore Sigma) was used according to the manufacturer’s protocol. Prism-GraphPad software was used to analyze data.

### Cell cycle analysis

Cells were treated with SP-1-303 for 24, 48, and 72 hours. DMSO and ionizing radiation (IR) were used on controls. The cell cycle distributions were analyzed by flow cytometry [22]. The cells were harvested at the indicated times, washed twice with cold phosphate-buffered saline (PBS), and fixed with citrate/DMSO buffer for overnight. The cells were resuspended in PBS containing 40 μg/ml of DNase-free RNase A and 50 μg/ml of propidium iodide, and the cell cycle distributions were measured in a fluorescence-activated cell sorter (FACSort) from Becton Dickinson. DNA content was quantified using ModFit LT 3.0 software (Verity Software House, Inc).

### Radiation clonogenic survival assay

Logarithmically growing cells were treated with vehicle (0.1% Dimethyl Sulfoxide) or SP-1-303 (0.3 μM) for 24 h followed by sham or γ-radiation using a Mark-30 irradiator with a 137Cs source at a fixed dose rate of 2.71 Gy/min. Cells were exposed to graded doses at room temperature. After 10–14 days, cells were fixed and stained with crystal violet, and colonies were counted. The surviving fractions of treated cells were normalized using the plating efficiencies of untreated controls. Radiation survival curves were generated by fitting to the single-hit, multitarget model [23].

### Western Blot and immunofluorescent analysis

Following treatment of cells with SP-1-303 over a time course, total or nuclear proteins were extracted and analyzed on 4–20% trisglycine gradient sodium dodecyl sulfate polyacrylamide gel electrophoresis (SDS-PAGE). Subsequently, membranes were probed with antibodies targeting specific proteins in accordance with the manufacturers’ protocols, including ATM (ab78), p-BRCA1 (ab90528), BRCA1 (ab191042), p-p53/S15 (ab1431), acetylated H3K9 (ab12179) from Abcam, as well as p-ATM (# 5883) and p53 (#9282) from Cell Signaling, α-tubulin (T6793) and β-actin (A3854) from Sigma-Aldrich, ER-α (cat# MA1-80216, Invitrogen) and HDAC2 (BML-SA402. Enzo). Protein images were captured using ECL Prime Western Blotting Detection Reagents (Cat#. RPN2232, Amersham) and the Amersham Imager 600. For immunofluorescent analysis, formalin fixed cells were permeabilized with 0.1% Triton X-100 in TBS for 10 minutes at room temperature, followed by blocking with 1% Blocker BSA for 15 minutes at room temperature. Cells were then probed with a phospho-ATM monoclonal antibody (MA5-15185) and DyLight 488 goat anti-mouse IgG secondary antibody at room temperature. Actin was stained with DyLight 554 phalloidin, while nuclei were stained with 4’,6-diamidino-2-phenylindole dye (DAPI). Images were captured using a Nikon Epi Fluorescent microscopy.

### Pharmacokinetics

A commercial vendor (Medicilon, Inc.) performed the pharmacokinetic studies. Male Sprague-Dawley Rats (n = 8) were received from Sino-British SIPPR/BK Lab Animal Ltd., Shanghai, and 6 animals were placed on study. All studies were approved by the Institutional Animal Care and Use Committee (Protocol #: 19098–17002) of Medicilon Preclinical Research-Shanghai LLC, Pudong Shanghai 201200, P.R. China. The animals were allowed to acclimatize for at least 5 d before beginning the study. The procedure was performed under general anesthesia with 3–5% isoflurane for blood collections via jugular vein. All efforts were made to minimizing suffering and least stressful. The compound was dissolved in 5% DMSO + 40% PEG400 + 55% PBS to yield a final concentration of 2 mg/ml. The tolerated dose (20 mg/Kg) was administered via intravenous (IV) in rats. After IV administration, blood samples were collected via jugular vein at various time points (0, 1, 2, 4, 6, and 8 h) under general anesthesia with 3–5% isoflurane. Samples were then placed into tubes containing K2EDTA and centrifuged at 8,000 rpm for 6 min to obtain plasma. The resulting plasma were analyzed by LCMS. Following completion of the study, all surviving animals were euthanized with CO2, followed by cervical dislocation. Pharmacokinetic analysis was determined using a non-compartmental module of WinNonlin Professional 5.2. The maximum measured plasma concentrations (Cmax) and the time of Cmax (tmax) were derived directly from the data. The area under the curve (AUC) was calculated using the log-linear trapezoidal method rule up to the last data point and was extrapolated to infinity with the following formula: Clast/β, where Clast is the last measured concentration time point. The elimination half-life was calculated with the equation t1/2 = 0.693/β, where β is the terminal rate constant. The clearance (Cl = dose/AUC) rate was also evaluated.

### Data analysis

All data were subjected to statistical analysis using Microsoft Excel. The program GraphPad Prism 10.2.2 was used to analyze data by construction of graphs and histograms.

## Results

### Design of dual-function small molecules

**Fig 1:**
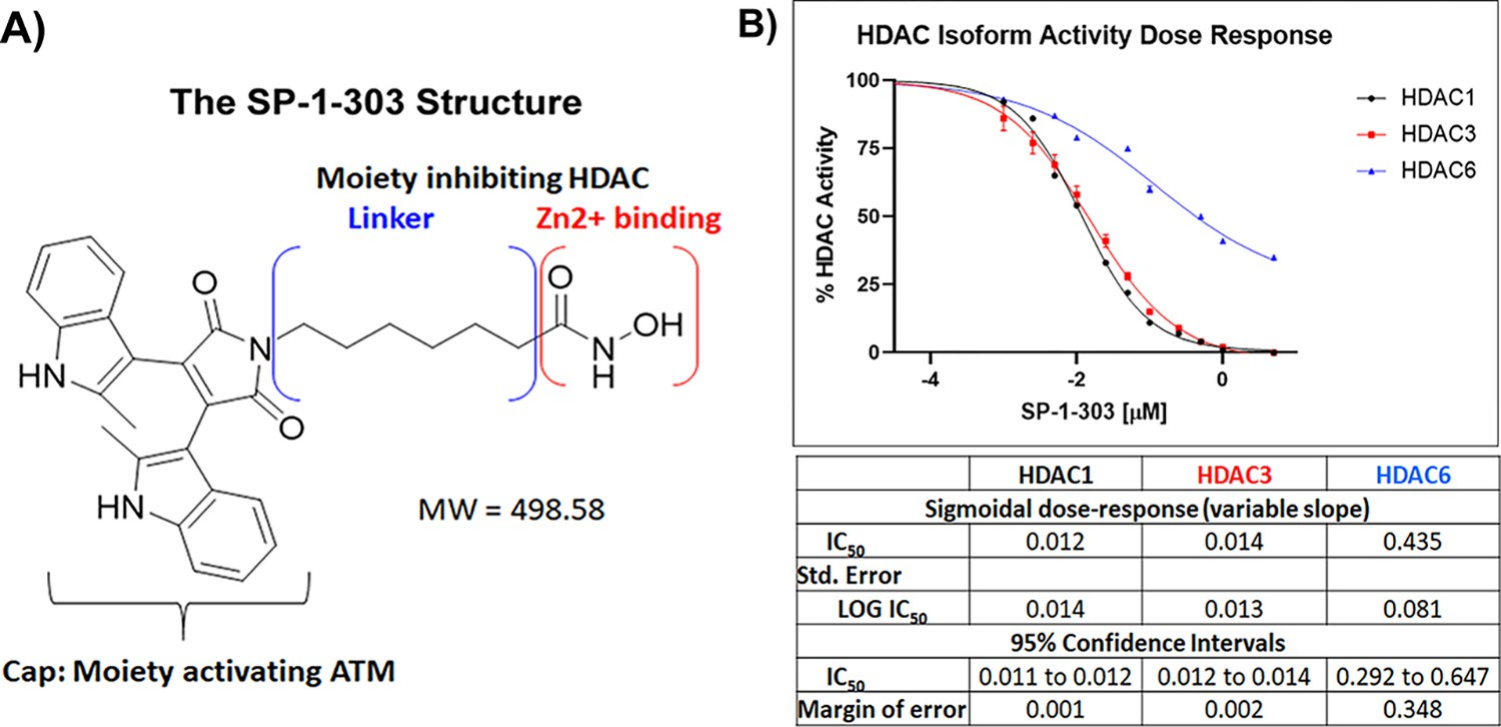
The SP-1-303 structure and *in vitro* HDAC isoform activity assays. **(A)** Schematic depiction of SP-1-303 with a molecular weight (MW) of 498.58. The design of the dual-targeting inhibitor, SP-1-303, employs a linked pharmacophore approach, combining the hydroxamate, pan-HDAC inhibitor domain with the 3,3’-Diindolylmethane (DIM) ATM activator domain. **(B)** *In vitro* HDAC isoform activities of HDAC1 (black squares), HDAC3 (red triangles), and HDAC6 (blue triangles), were analyzed to calculate EC_50_ values with error bars and 95% confidential intervals with the margin of error using Prism-GraphPad.

The design of dual-function small molecules was achieved through a rational design and synthesis utilizing a merged pharmacophore methodology and screening for ATM activation and HDAC inhibition, leading to the discovery and development of the dual-targeting inhibitor, SP-1-303. The bi-functional molecule was synthesized by merging the hydroxamate (pan-HDAC inhibitor SAHA) with the 3,3’-Diindolylmethane (the ATM activation domain for the Cap of the molecule), forming a compound capable of simultaneously affecting two target molecules (Fig 1A). In the design process, we considered two indole structures with two methyl groups (dimethyl-di-indoles) to develop a second generation HDAC inhibitor, aiming to target histone deacetylases and the DNA damage sensor ATM [24].

Our focus centered on the inhibition activities of HDAC1 and HDAC3, class I HDACs pivotal in cell proliferation, and on the class II HDAC6, pivotal in regulation of the innate immune system. A panel of purified HDAC enzymes was utilized to delineate isoform specificity by testing pan-HDACs, HDAC1, HDAC3, and HDAC6 *in vitro* in a cell-free system. SP-1-303 exhibited specificity in inhibiting C HDAC1 and HDAC3, preferentially to HDAC6 (Fig 1B).

The data in Table 1 summarizes *In vitro* HDAC enzyme inhibition activities of SP-1-303 and SAHA utilizing HeLa cell extract for pan-HDACs and purified recombinant proteins for HDAC1, HDAC3, and HDAC6. The IC_50_ values of SP-1-303 for HDAC1 and HDAC3 activity inhibition are at 12 nM and 14 nM, respectively. The IC_50_ values of SP-1-303 for pan-HDACs and the class II HDAC6 inhibition are at 120 nM and 435 nM, respectively.

**Table 1:** Inhibition of pan- and HDAC isoform activities.

	IC_50_ (nM) HDAC inhibition activity *in vitro*				
Compound	Pan-HDAC	HDAC 1	HDAC 3	HDAC 6	Ratio (HDAC6:HDAC1)
SAHA	36±7	40.6±8	46.5±6	17.7±4	0.4
SP-1-303	120±58	12±1	14±2	435±35	36

**Note:** *In vitro* HDAC enzyme inhibition assays utilized HeLa cell nuclear extract for pan-HDACs and purified recombinant proteins for HDAC1, HDAC3, and HDAC6. The IC_50_ values with the margin of error (±) of 95% confidential intervals were analyzed by Prism-GraphPad.

The ratio of inhibitory concentrations for HDAC6 to HDAC1 was 36-fold. In addition, the IC_50_ values are at 0.31 μM for HDAC8 activity inhibition and at > 20 μM for class IIa HDAC4 and HDAC5 inhibition activities (data not shown). The SAHA control demonstrated pan-HDAC inhibition activity at 36 nM and HDAC6 inhibition at 17.7 nM. Together, these data support the selective inhibitor, SP-1-303, as targeting Class I HDAC1 and HDAC3.

**Fig 2:**
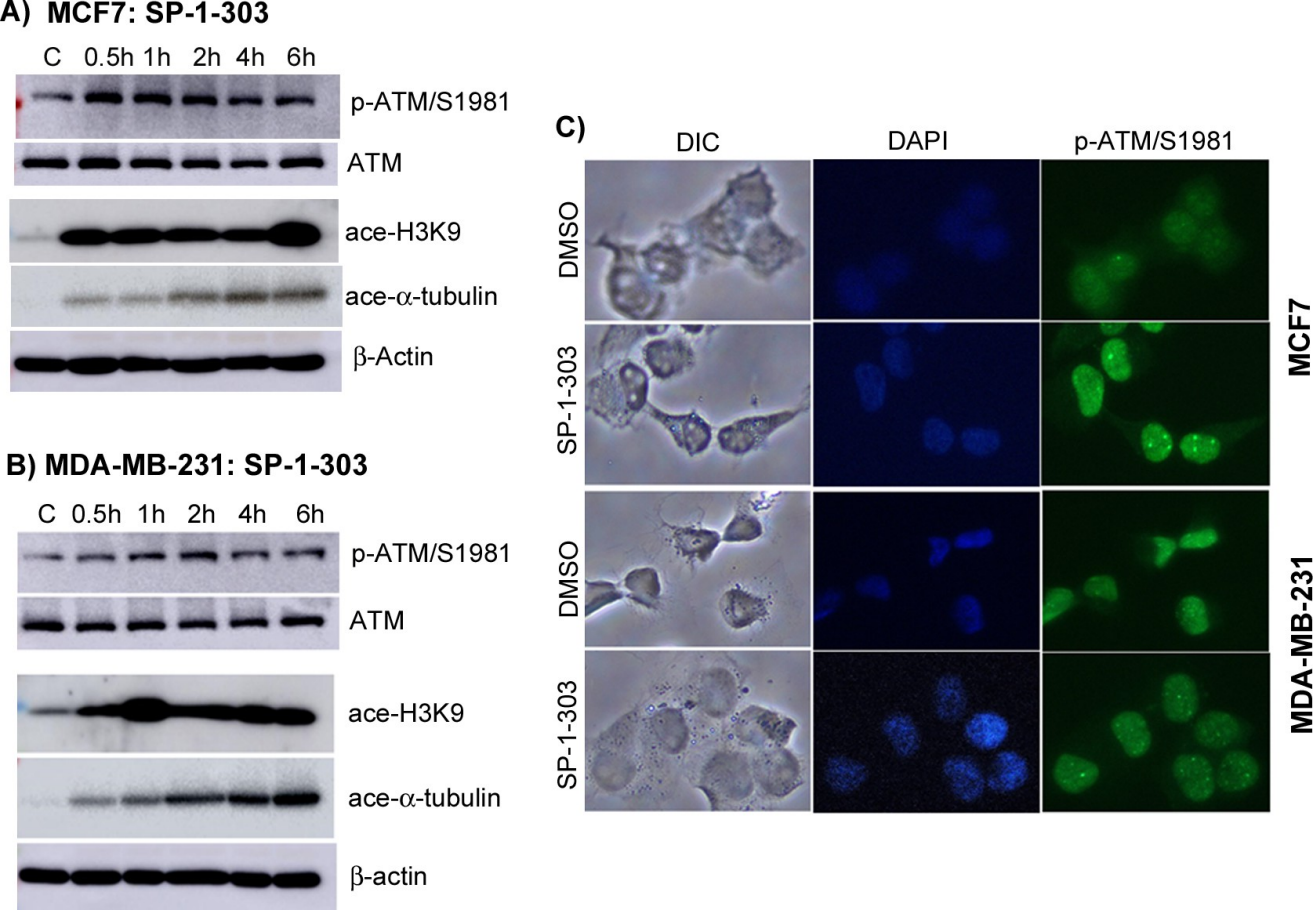
Analysis of dual-functional activity of SP-1-303. Western blot analyses of total proteins were conducted to assess the expression levels of p-ATM/S1981, total ATM (~350 kDa), acetylated histone 3 lysine 9 (H3K9ac) (17 kDa) and acetylated α-tubulin (55 kDa) in MCF7 **(A)** and MDA-MB-231 **(B)** cells post-treatment with SP-1-303 (1 μM) over a time course, with DMSO (0.05%) serving as a control. β-Actin (42 kDa) was utilized to ensure equal loading of protein. **(C)** Immunofluorescent analyses of p-ATM (green) in both MCF7 and MDA-MB-231 cells were performed subsequent to treatment with either vehicle (DMSO) or 1 μM SP-1-303 for 4 hours. Cells were probed with DAPI for nuclei staining, and a p-ATM antibody subsequently incubated with DyLight 488 goat anti-mouse IgG secondary antibody at room temperature. Images were captured using Nikon Epi Fluorescence microscopy at 20X magnification and differential interference contrast (DIC) microscopy for contrast.

The dual-function of SP-1-303 was further explored by assessing the acetylation of two common targets, histone 3 (H3) and 〈-tubulin, as well as the phosphorylation of ATM in both MCF7 and MDA-MB-231 cells. Notably, SP-1-303 treatment exhibited significantly enhanced acetylation of histone H3 lysine 9 (H3K9) and phosphorylation of ATM/S1981 within 0.5 h of cell exposure. Moreover, the acetylation of α-tubulin was gradually increased in a time dependent manner (Fig 2A & 2B). We hypothesized that the observed α-tubulin acetylation may result from potential off-target activities due to the higher drug concentrations utilized in cellular studies. Collectively, these findings substantiate SP-1-303’s dual-functionality as a molecule capable of inhibiting HDAC activities while activating ATM via phosphorylation. Immunocytochemical analysis further supports ATM activation following a 4hour treatment, as evidenced by pronounced foci in the nucleus, albeit more prominently observed in MCF7 cells (Fig 2C).

### The effect of SP-1-303 on breast normal and cancer cell growth

Inhibition of cancer cell growth with little effect on surrounding normal cells is the desired strategy for treatment of cancers. The half maximal effective concentrations (EC_50_) of SP-1-303 were determined in four breast cancer cell lines (two ER+, MCF7 and T47D; two TNBC, HCC-1937 and MDA-MB-231) and one normal breast epithelial cell line (MCF10A) (Table 2).

**Table 2:** Effective cell growth inhibition of drugs in breast normal and cancer Cells.

Cell Line	Compound	Average
		EC_50_ [μM] ± SD
MCF10A (Normal)	SAHA	10.130±0.982
	SP-1-303	12.168±3.596
	Tamoxifen	14.955±0.233
MCF7 (ER^+^)	SAHA	3.649±0.151
	SP-1-303	0.340±0.122
	Tamoxifen	1.582±0.511
T47D (ER^+^)	SAHA	3.902±0.469
	SP-1-303	0.321±0.027
	Tamoxifen	1.691±0.218
MDA-MB-231 (TNB)	SAHA	2.412±1.506
	SP-1-303	1.220±0.243
	Tamoxifen	15.350±1.853
HCC-1937 (TNB)	SAHA	11.340±1.867
	SP-1-303	2.483±0.280
	Tamoxifen	1.494±0.213

**Note**: The average of the half maximal effective concentration (EC_50_) and the mean of standard error deviation (±SD) of each compound were obtained from the dose response curves of at least two to three independent experiments. Cells were treated with compounds for 48 h.

**Fig 3:**
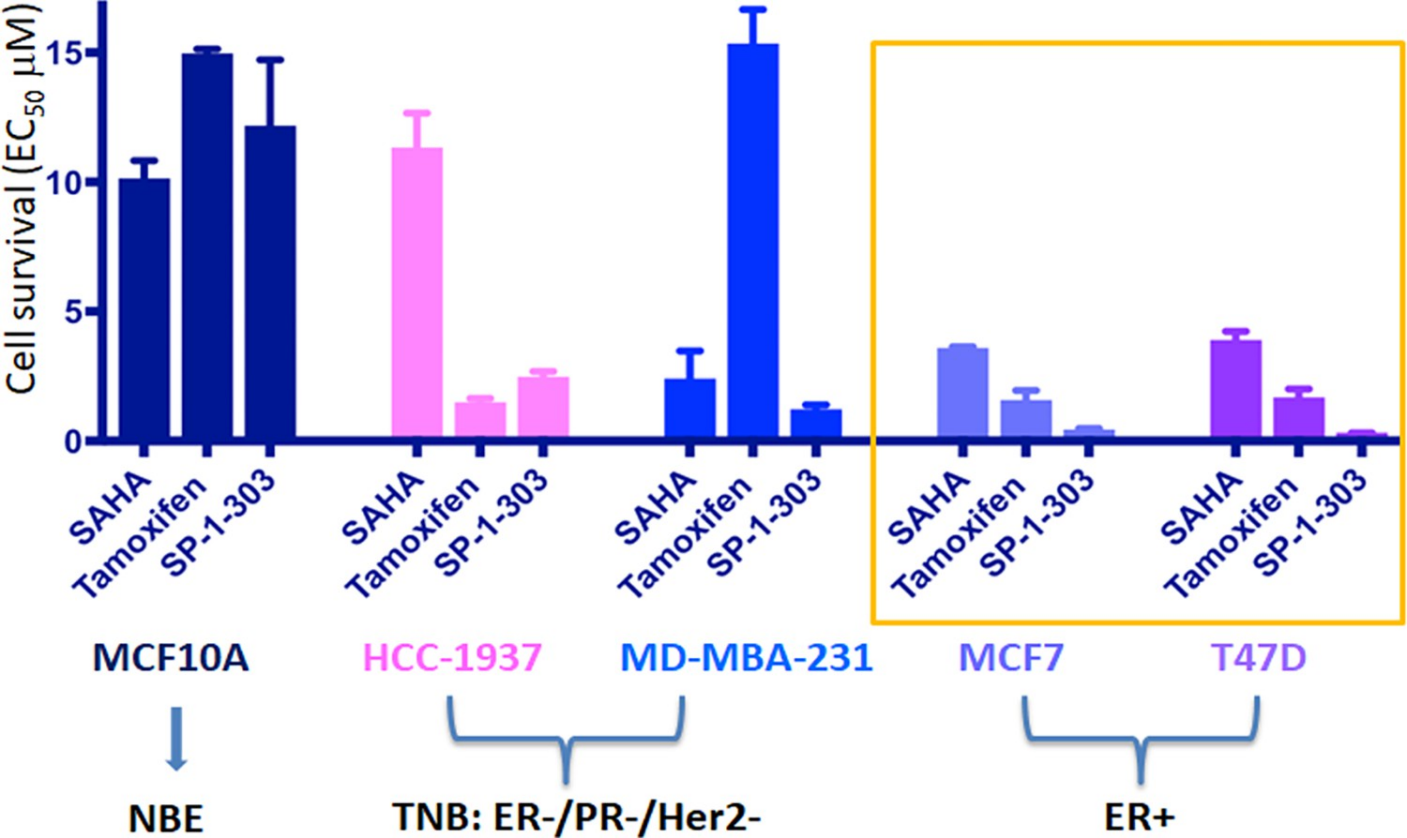
Growth inhibitory efficacy of drugs in normal breast epithelial and cancer cells. Comparison of growth inhibition effects of three drugs (SAHA, Tamoxifen, and SP-1-303) in various breast cell lines, including ER+ BC, TNBC, and normal breast epithelial cell lines. The ER+ BC cell lines (MCF7 and T-47D), two triple negative breast cancer (TNBC) cell lines (MDA-MB-231 and HCC 1937), and one normal breast epithelial cell line (MCF10A) were tested to determine the concentration inhibiting cell proliferation by 50% effective response (EC_50_) with the standard error (+/-) of each compound.

The effect of SP-1-303 on cell growth was dose-dependent. MCF7 and T47D had the lowest EC_50_s, 0.34 μM and 0.32 μM, respectively, compared to ER negative TNBC cell lines (2.5 μM for HCC-1937, 1.2 μM for MA-MB-231) and one normal breast cell line (12.2 μM for MCF10A). The data demonstrated that SP-1-303 preferentially inhibited ER+ breast cancer growth. We attribute these growth inhibitory effects on the ER+ cancer cells in the presence of the di-indole, di-methyl structure. The efficacy of SP-1-303 in ER+ breast cancer cell growth was further compared to other drugs, SAHA and Tamoxifen, in normal, ER+, and TNBC cells. Both ER+ breast cancer cells (MCF7 and T47D) revealed significant sensitivity to SP-1-303 as compared to SAHA and Tamoxifen (Table 2 & Fig 3).

EC_50_ values were an average of at least two to three independent experiments. Dose response curves representing the efficacy of SP-1-303, SAHA, and Tamoxifen, along with 95% confidence intervals and margins of error, in each cell line were shown in S1–S5 Figs. It is clear that ER+ breast cancer cells are more sensitive to SP-1-303 than are cells from other breast cancer types. SAHA and SP-1-303 conferred EC_50_ at 2.4 μM and 1.22 μM, respectively, in MDA-MB-231, while Tamoxifen conferred low sensitivity similar to MCF10A. Furthermore, EC_50_ values of SP-1-303 and Tamoxifen were at 2.48 μM and 1.49 μM, respectively, in HCC-1937, but the EC_50_ value of SAHA was similar to that observed in MCF10A. Comparing the HDAC inhibitors, SP-1-303 was 10-fold more potent in ER+ cell lines than is SAHA. All tested drugs demonstrated significantly lower growth inhibition in normal breast epithelial cells. Our findings are interpreted to show that SP-1-303 exhibits toxicity preferentially against ER+ breast cancer cells when compared to normal breast epithelial cells (MCF10A) and triple-negative breast cancer cells (HCC1937 and MDA-MB-231). This specificity stands in contrast to SAHA, a pan-HDAC inhibitor. These results underscore SP-1-303 as a promising candidate for further evaluation as a drug specifically targeting ER-positive breast cancer.

### ER-α and potential targets regulated by SP-1-303

**Fig 4:**
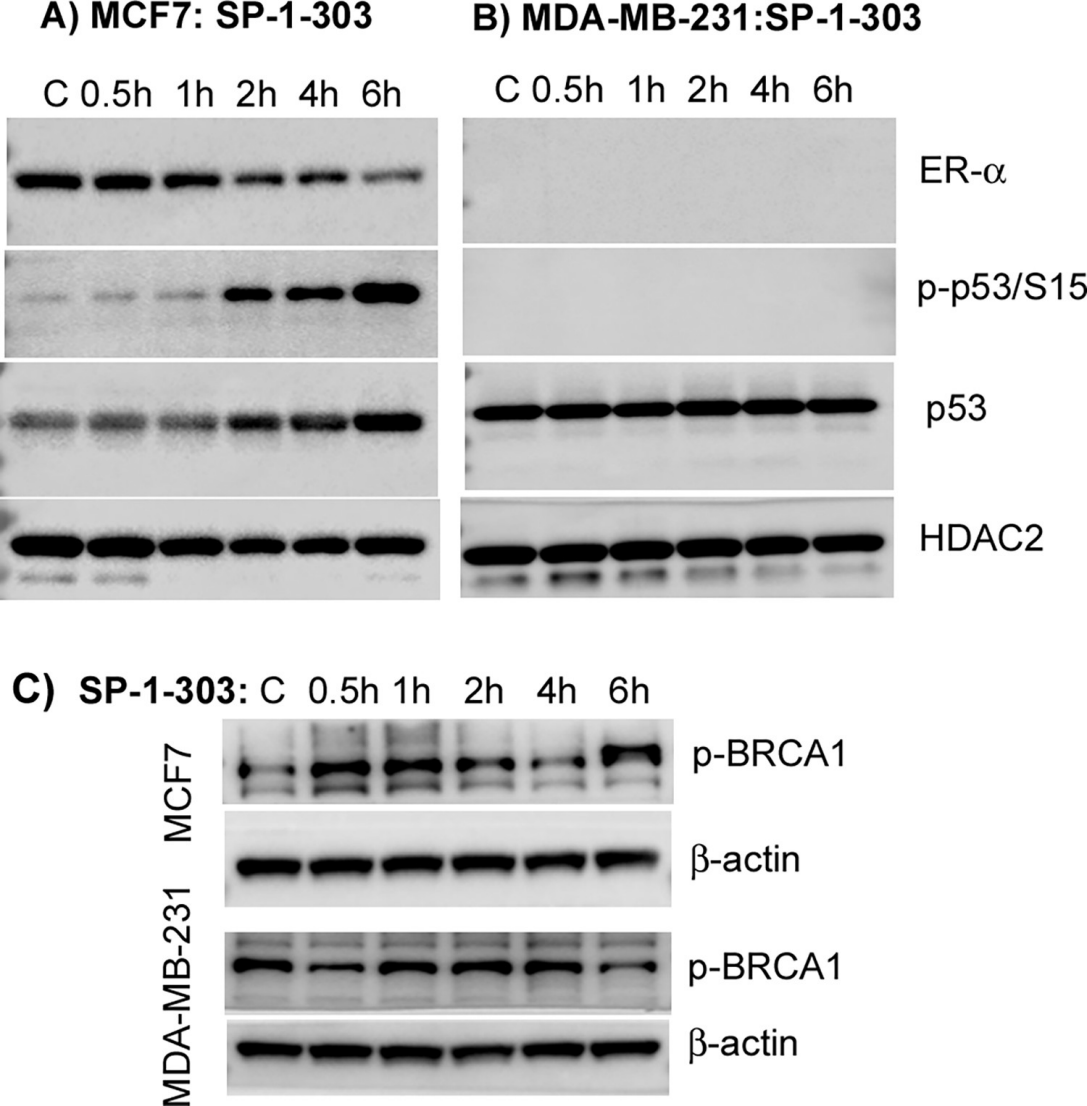
The impact of SP-1-303 on target molecules. Following treatment of cells with SP-1-303 over a time course, nuclear extracts were analyzed on 4–20% trisglycine gradient gels. **(A)** The expression levels of ER-α (~66 kDa) and p53 (53 kDa) were evaluated in MCF7 following treatment with SP-1-303 (1 μM) over a time course. DMSO (0.05%), a vehicle, serves as a control treatment. The HDAC2 protein expression level with no changes serves as a loading control. **(B)** The expression levels of ER-α and p53 were examined in MDA-MB-231 following treatment with SP-1-303 (1 μM) over a time course. DMSO (0.05%) serves as a control treatment. HDAC2 (55–60 kDa) serves as a loading control. **(C)** The phosphorylated BRCA1 (~220 kDa) levels were analyzed in both MCF7 and MDA-MB-231 cells following treatment with 1μM SP-1-303 or DMSO as a control for the indicated durations. β-actin (42 kDa) serves as a loading control.

To elucidate the mechanism of action specific to ER+ breast cancer cells, we investigated the expression levels of ER-α protein following treatment of MCF7 and MDA-MB-231 cells with SP-1-303 over a time course of 0.5h to 6h (Fig 4).

The data reveals a gradual reduction in ER-α expression from 2h to 6h in MCF7 cells (Fig 4A), while there is no detected expression in MDA-MB-231 (Fig 4B), consistent with previous studies, which demonstrate that pan-HDAC inhibitors, such as TSA and SAHA, can repress ERα expression and inhibit the growth of ER-positive breast cancer cells [25]. Conversely, our data shows that the expression of p53 exhibits a gradual increase in MCF7 cells following treatment with SP-1-303, with no significant change observed in MDA-MB-231 cells. Additionally, we examined the impact of SP-1-303 on the phosphorylation of BRCA1 and p53, ATM substrates, and observed an induction of phosphorylated BRCA1 and p53 within 1h to 2h (Fig 4A & 4C). Notably, phosphorylated BRCA1 without significant alteration and no phosphorylated p53 were detected in MDA-MB-231 (Fig 4B & 4C). These findings suggest that the activation of cellular pathways involves the DNA damage response and apoptosis, which could confer advantages in inhibiting cancer cell growth.

### SP-1-303 induces G1 arrest of cell cycle

**Fig 5:**
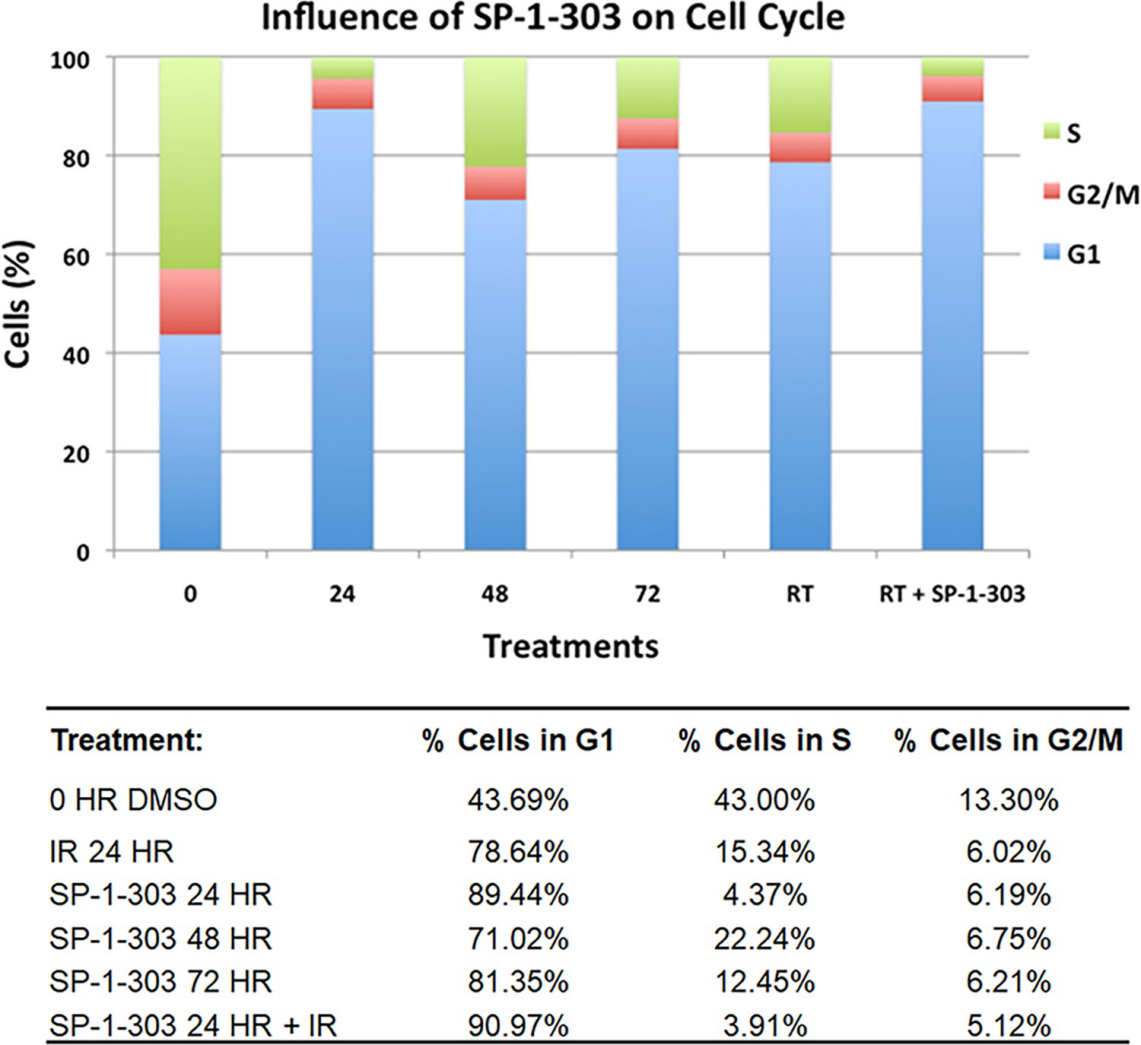
SP-1-303 induces G1 arrest. Flow cytometry (FACS) analyses were performed to measure changes in cell cycle distribution after treatments. MCF7 cells were treated with SP-1-303 for 24, 48, and 72 hours, DMSO and radiation (IR) were used as controls. In addition, cells were treated with a combination of radiation and SP-1-303 after 24 hours. DNA content was quantified using ModFit LT 3.0 software (Verity Software House, Inc).

To understand the growth inhibition mechanism of SP-1-303, the cell cycle distribution of MCF7 cells was analyzed by flow cytometry (FACS). The data demonstrate significant increases in G1 arrest (81–89%) within 24 h– 72 h and in the G1 phase (91%) of the cell cycle following drug treatment in combination with radiation (RT) (Fig 5). This observation supports a drug mechanism leading to growth arrest in the G1 phase of the cell cycle after treatment. In addition, G1 phase arrest is in a radiation sensitive phase, suggesting SP-1-303 synergism with radiation on cell killing.

### Radiation survivals

**Fig 6:**
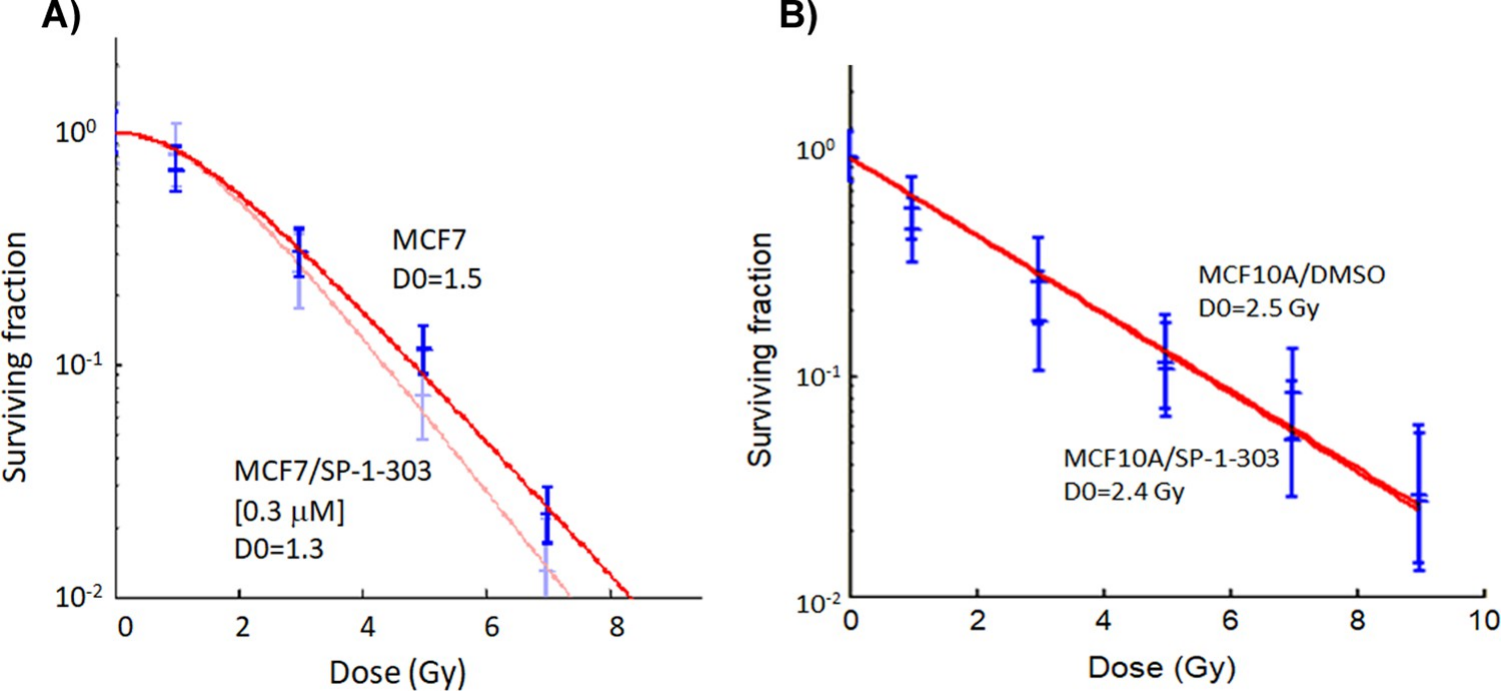
Radiation survival assay. Logarithmically growing MCF7 and MCF10A cells were treated with SP-1-303 (0.3 μM) for 24 h followed by graded doses of γ-radiation (IR). After 10–14 days, colonies were fixed, stained with crystal violet, and counted. The surviving fractions of treated cells were normalized using the plating efficiencies of untreated controls. Radiation survival curves were fitted by computer to the single-hit, multitarget model.

Cells in the G1 and G2/M phases of cell cycle are more sensitive to radiation-mediated injury than in other phases. Repair of DNA damage by HR and NHEJ mechanisms occurs predominantly in the G1 and G2/M. To examine the radiation sensitizing effect of SP-1-303 treatment, radiation clonogenic survivals were determined after exposure of cells to SP-1-303 for 24h followed by exposure to graded doses of radiation. The D_0_ values were at 1.5 Gy for sham controls treated with DMSO and 1.3 Gy for cells treated with Sp-1-303 (0.3 μM). MCF10A cells exhibited little effect on the control (2.5 Gy) and the drug treated cells (2.4 Gy) (Fig 6). Together, the data demonstrate that SP-1-303 enhances radiation cell killing of MCF7 cells while conferring little effect on normal breast epithelial cells, MCF10A.

### Pharmacokinetic (PK) analysis of SP-1-303

**Fig 7:**
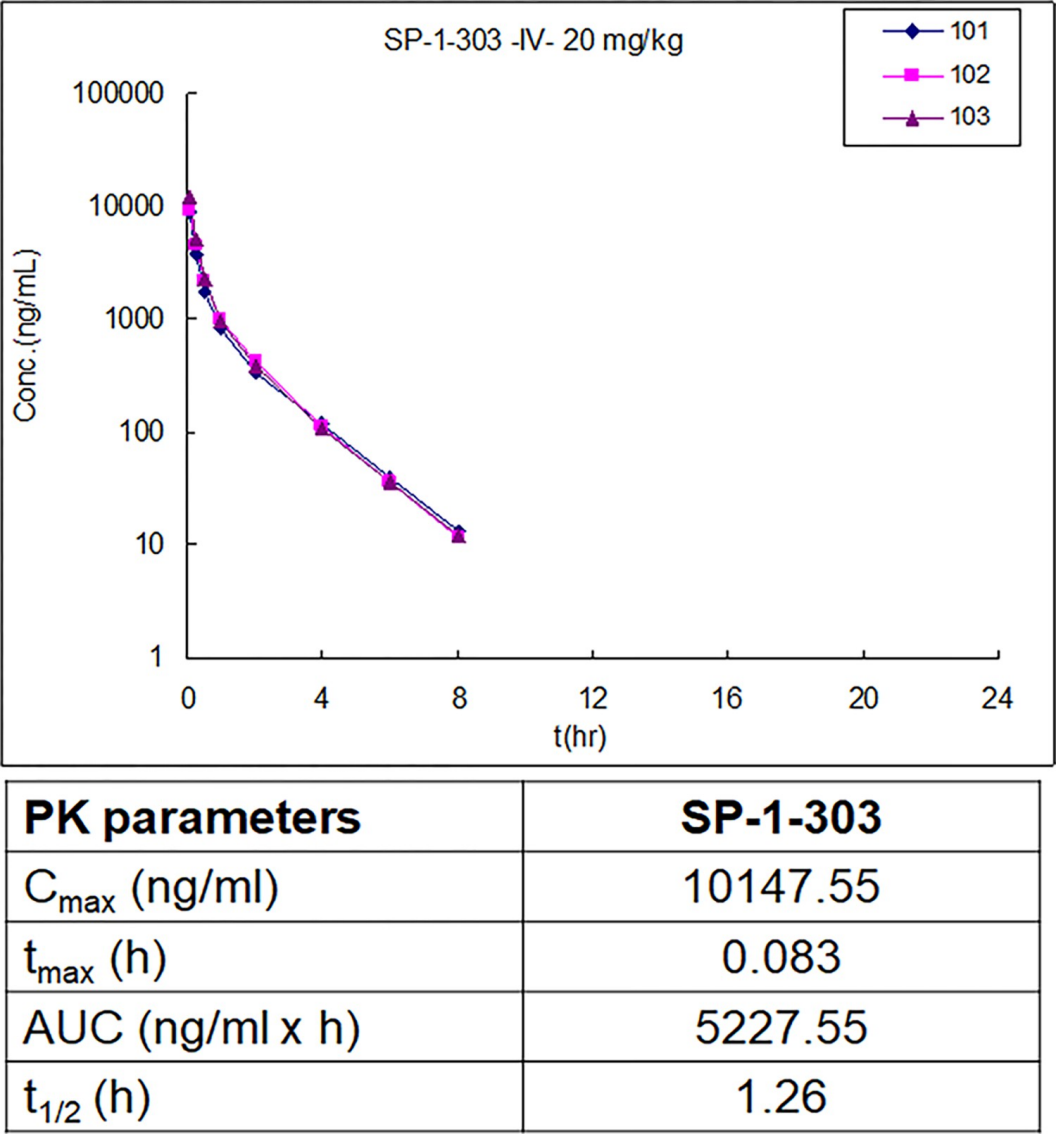
Pharmacokinetics of SP-1-303. The compound was administered intravenously at 20 mg/kg in Sprague-Dawley rats (n = 3). Selected PK parameters were determined at various time points (0-8hrs) in blood samples. Pharmacokinetic parameters are as follows: C_max_, the maximum plasma concentration; t_max_, the time to reach maximum plasma concentration; AUC, the area under the compound concentration versus time curve; t_1/2_, the elimination half-life.

To translate the *in vitro* properties of SP-1-303 to *in vivo* studies, the preliminary PK of SP-1-303 was determined. The compound was administered at 20 mg/kg via intravenous (IV) in Sprague-Dawley rats. The plasma PK of SP-1-303 was then determined up to 8h (Fig 7). The data show that SP-1-303 is absorbed and eliminated rapidly with a t_max_ = 0.083 (mean value) and a half-life (t_1/2_) = 1.26h in rats. 1% remained at 4h. The maximum plasma concentration (Cmax) was 0147.55 ng/ml, and the area under the curve was 5227.55 x h.

## Discussion

Our findings characterize SP-1-303 as a novel, second generation, class I HDAC1 and HDAC3 selective HDAC inhibitor, exhibiting ATM activation and modulation of ER expression, resulting in substantial growth inhibition of ER+ BC cells. Notably, SP-1-303 demonstrates significantly reduced toxicity to normal breast epithelial cells (40-fold lower toxicity compared to cancer cells). These results are consistent with previous studies indicating that pan-HDAC inhibitors, such as TSA and SAHA, can downregulate both mRNA and protein levels of ERα, thereby inhibiting the growth of ER-positive breast cancer cells like MCF7 and T47D cells [25–27]. Furthermore, the overexpression of HDAC1 has been linked to negative regulation of ER-α, suggesting a potential role for HDACs in modulating breast cancer progression by regulating ER-α levels and activity. Supporting these findings, our data reveal a gradual increase in p53 and BRCA1 phosphorylations in MCF7 following treatment with SP-1-303, while no significant changes were observed in MDA-MB-231. Additionally, cell cycle analysis demonstrates that SP-1-303 induces G1 arrest in MCF7 cells within 24 hours of exposure. Moreover, the G1 arrest is additive to the cell cycle arrest induced by ionizing radiation, suggesting potential application in combination with radiation therapy. Together, these data demonstrate SP-1-303 as a highly potent inhibitor of ER+ breast cancer cell growth, with minimal effects on TNBC and normal breast epithelial cells. Preclinical pharmacokinetic analysis reveals a half-life (t_1/2_) of 1.26 hours in rats.

Roles for specific HDAC isoforms in cell cycle regulation, DNA damage responses, and radiation sensitivity have been reported [28–31]. We reported that ATM interacts with HDAC1, suggesting HDAC involvement in ATM regulation [32]. Others have confirmed ATM’s influence on histone-mediated gene regulation by using HDAC inhibitors [33,34] and have observed HDAC4 co-localization with 53BP1 in the DNA damage response [35]. These reports provide both direct and indirect evidence for HDAC isoforms playing multiple roles in DNA damage response networks, and that HDAC inhibitors are capable of sensitizing cancer cells to killing by ionizing radiation. Our observation that the sensitivity of cancer cells to ionization radiation is increased in the presence of HDAC inhibitors [36–38] further supports chromatin modification in the mechanism underlying SP-1-303 cytotoxic effects.

First generation hydroxamic acid based HDAC inhibitors and one cyclic depsipeptide have been approved by the FDA for the treatment of hematological malignancies (cutaneous T-cell lymphoma, peripheral T-cell lymphoma, multiple myeloma), including Vorinostat (a.k.a. SAHA; hydroxamic acid), Belinostat (PXD-101; hydroxamic acid), Panobinostat (LBH589; hydroxamic acid) and Romidepsin (FK-228; cyclic depsipeptide). Panobinostat’s FDA approval for refractory multiple myeloma and Romidepsin for the PTCL indications have been withdrawn due to lack of clinical feasibility [10,11]. With observed lack of success of pan-HDAC inhibitors, combination approaches with other anti-cancer agents are emerging [39]. Panobinostat was approved in combination with bortezomib and dexamethasone in patients with recurrent multiple myeloma. Other studies have also shown that combinations of panobinostat with an EGFR inhibitor suppressed claudin-low (CL) TNBC cell proliferation, proposing class I HDACs as therapeutic targets for breast cancer patients with TNBC [40]. Entinostat (benzamide MS-275) was reported as a class I inhibitor and underwent clinical trials with other agents for ER+ BC treatment [17,18]. However, the combination of entinostat with exemestane did not improve survival in aromatase inhibitor resistant hormone receptor positive, HER2-negative breast cancers [19].

Despite attempts to combine HDAC inhibitors with conventional therapeutic agents, success has been limited due to issues that include drug resistance, low efficacy, dose-limiting toxicities, and side effects [41]. Currently, dual-targeting molecules have been developed as single molecules by combining pan-HDAC inhibitors with target inhibitors, such as Topo I/II, PARP, or others [41]. Interestingly, these dual function molecules exhibit higher inhibitory activities against specific HDACs [42,43], consistent with our observations. However, in contrast to such dual function inhibitors, SP-1-303 has the unique property by activating ATM while inhibiting HDACs, selectively targeting HDAC1 and HDAC3. Therefore, this molecule warrants further investigation.

While ATM is widely recognized as a tumor suppressor and plays a crucial role in DNA damage sensing and response, some cancer cells can exploit ATM activation to develop resistance to chemotherapy by activating ATM. Blocking ATM activation aims to sensitize cancer cells to chemotherapy, but this approach may result in toxicities to healthy cells that rely on ATM for DNA repair and affect numerous ATM substrates, including tumor suppressors such as Brca1 and p53. Therefore, overcoming ATM-related drug resistance presents a challenge. The ability of our dual-targeting SP-1-303 to gradually increase p53 protein expression suggests it may activate cellular pathways involved in DNA damage response and apoptosis, which could inhibit cancer cell growth. Additionally, phosphorylation of ATM and BRCA1 proteins further supports its involvement in DNA repair mechanisms. Overall, the compound’s dual effect offers therapeutic potential for breast cancer treatment by targeting both DNA repair pathways and hormone receptor signaling, particularly in ER-positive breast cancer cases.

Given that epigenetic changes affecting gene expression and overexpression of histone deacetylases (HDACs) in cancer commonly cause resistance to genotoxic-based therapies, our dual-target approach, combining an ATM activator with an HDAC inhibitor, may harness the benefits of ATM activation while enhancing chemotherapy effectiveness and minimizing resistance, potentially improving treatment outcomes. Recent findings that heterozygous carriers of *ATM* are at increased risk of developing breast cancer with poor prognosis, and that the *ATM* gene is among the commonly aberrant genes in human sporadic breast cancer [20,21], suggest that our dual-targeting SP-1-303 HDAC inhibitor linked with an ATM activator may offer a novel approach for breast cancer treatment. Further preclinical studies of SP-1-303 will determine its potential for breast cancer treatment.

## Supporting information

S1 Fig Effective cell growth inhibition (EC_50_ μM) of drugs in normal breast epithelial cells, MCF10A. Dose responses of (A) SP-1-303, (B) SAHA, and (C) Tamoxifen, were analyzed by Prism-GraphPad. The average percentage of cell growth was analyzed by the log concentration of EC_50_ values with corresponding 95% confidence intervals with margins of error.
(TIF)

S2 Fig Effective cell growth inhibition (EC_50_ μM) of drugs in ER positive MCF7 cells. Dose responses of (A) SP-1-303, (B) SAHA, and (C) Tamoxifen, were analyzed by Prism-GraphPad. The average percentage of cell growth was analyzed by the log concentration of EC_50_ values with corresponding 95% confidence intervals with margins of error.
(TIF)

S3 Fig Effective cell growth inhibition (EC_50_ μM) of drugs in ER positive T47D cells. Dose responses of (A) SP-1-303, (B) SAHA, and (C) Tamoxifen, were analyzed by Prism-GraphPad. The average percentage of cell growth was analyzed by the log concentration of EC_50_ values with corresponding 95% confidence intervals with margins of error.
(TIF)

S4 Fig Effective cell growth inhibition (EC_50_ μM) of drugs in TNB (ER negative) HCC-1937 cells. Dose responses of (A) SP-1-303, (B) SAHA, and (C) Tamoxifen, were analyzed by Prism-GraphPad. The average percentage of cell growth was analyzed by the log concentration of EC_50_ values with corresponding 95% confidence intervals with margins of error.
(TIF)

S5 Fig Effective cell growth inhibition (EC_50_ μM) of drugs in TNB (ER negative) MDA-MB-231 cells. Dose responses of (A) SP-1-303, (B) SAHA, and (C) Tamoxifen, were analyzed by Prism-GraphPad. The average percentage of cell growth was analyzed by the log concentration of EC_50_ values with corresponding 95% confidence intervals with margins of error.
(TIF)

S1 Raw images (PDF)
