# Supplementary figures and images for: Dual-targeting class I HDAC inhibitor and ATM activator, SP-1-303, preferentially inhibits estrogen receptor positive breast cancer cell growth
Source: PLoS One. 2024 Jul 15;19(7):e0306168. doi: 10.1371/journal.pone.0306168 (PMC11249239; doi:10.1371/journal.pone.0306168)

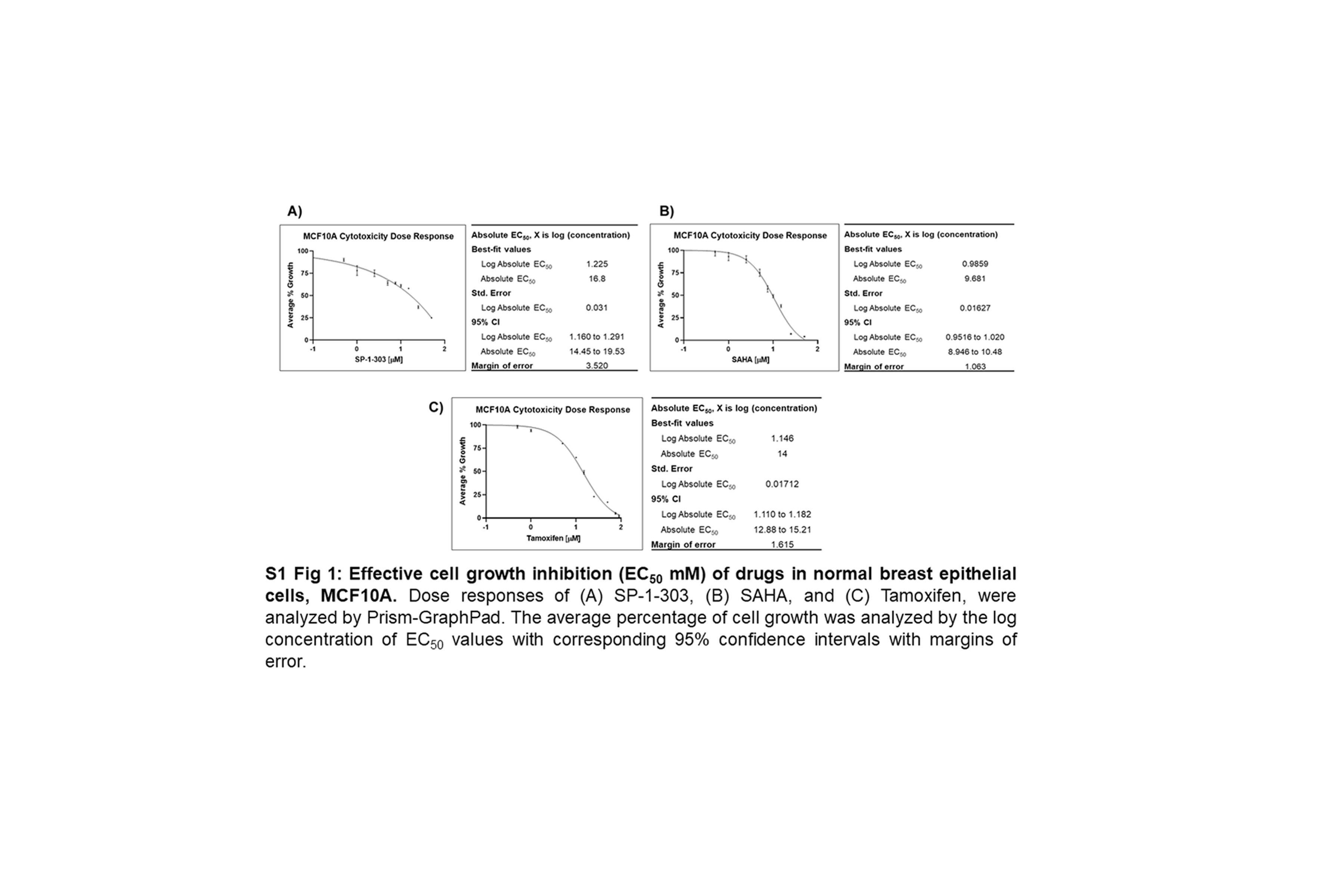

Supplement: S1 Fig — Dose responses of (A) SP-1-303, (B) SAHA, and (C) Tamoxifen, were analyzed by Prism-GraphPad. The average percentage of cell growth was analyzed by the log concentration of EC50 values with corresponding 95% confidence intervals with margins of error. (TIF) [file pone.0306168.s001.tif]

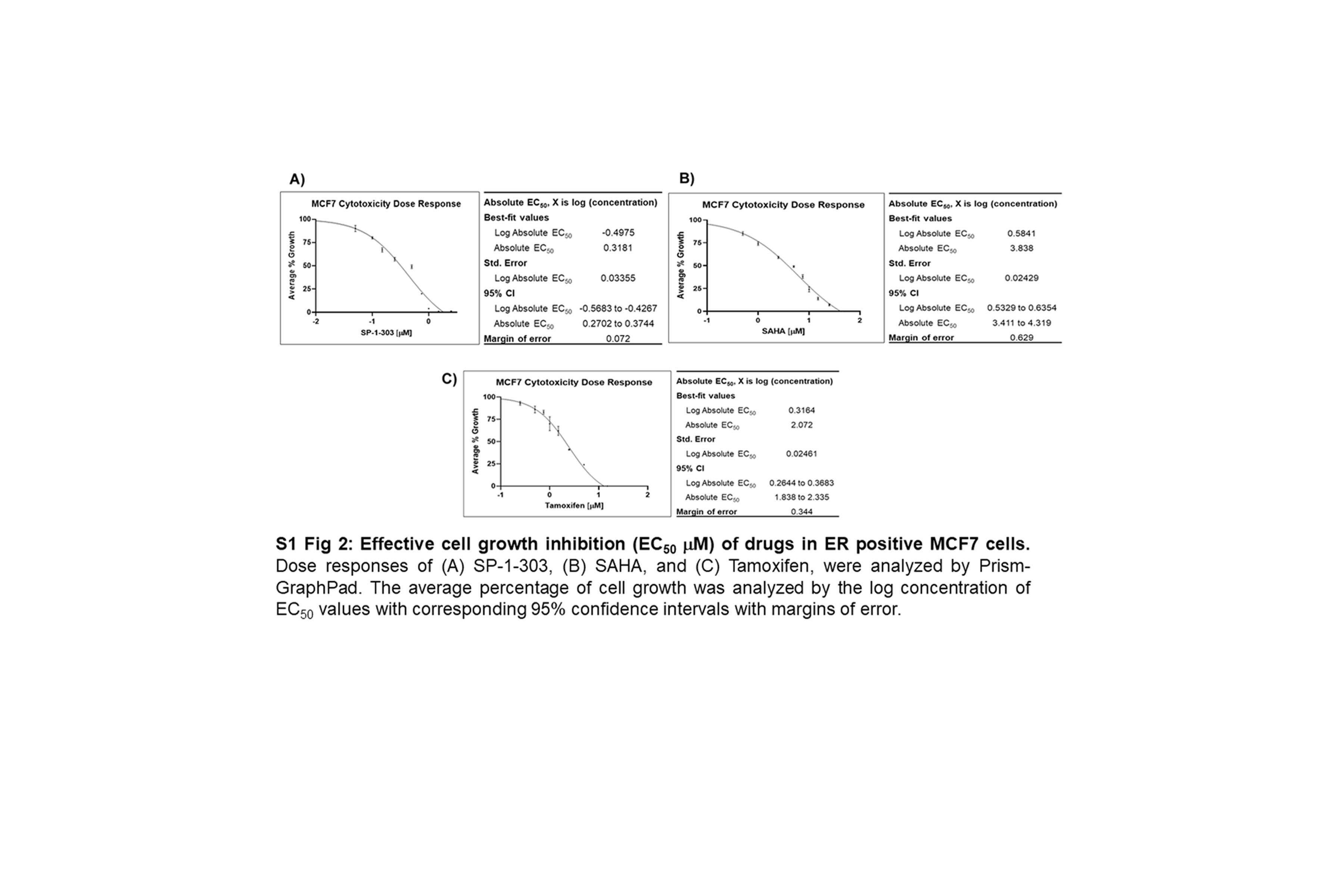

Supplement: S2 Fig — Dose responses of (A) SP-1-303, (B) SAHA, and (C) Tamoxifen, were analyzed by Prism-GraphPad. The average percentage of cell growth was analyzed by the log concentration of EC50 values with corresponding 95% confidence intervals with margins of error. (TIF) [file pone.0306168.s002.tif]

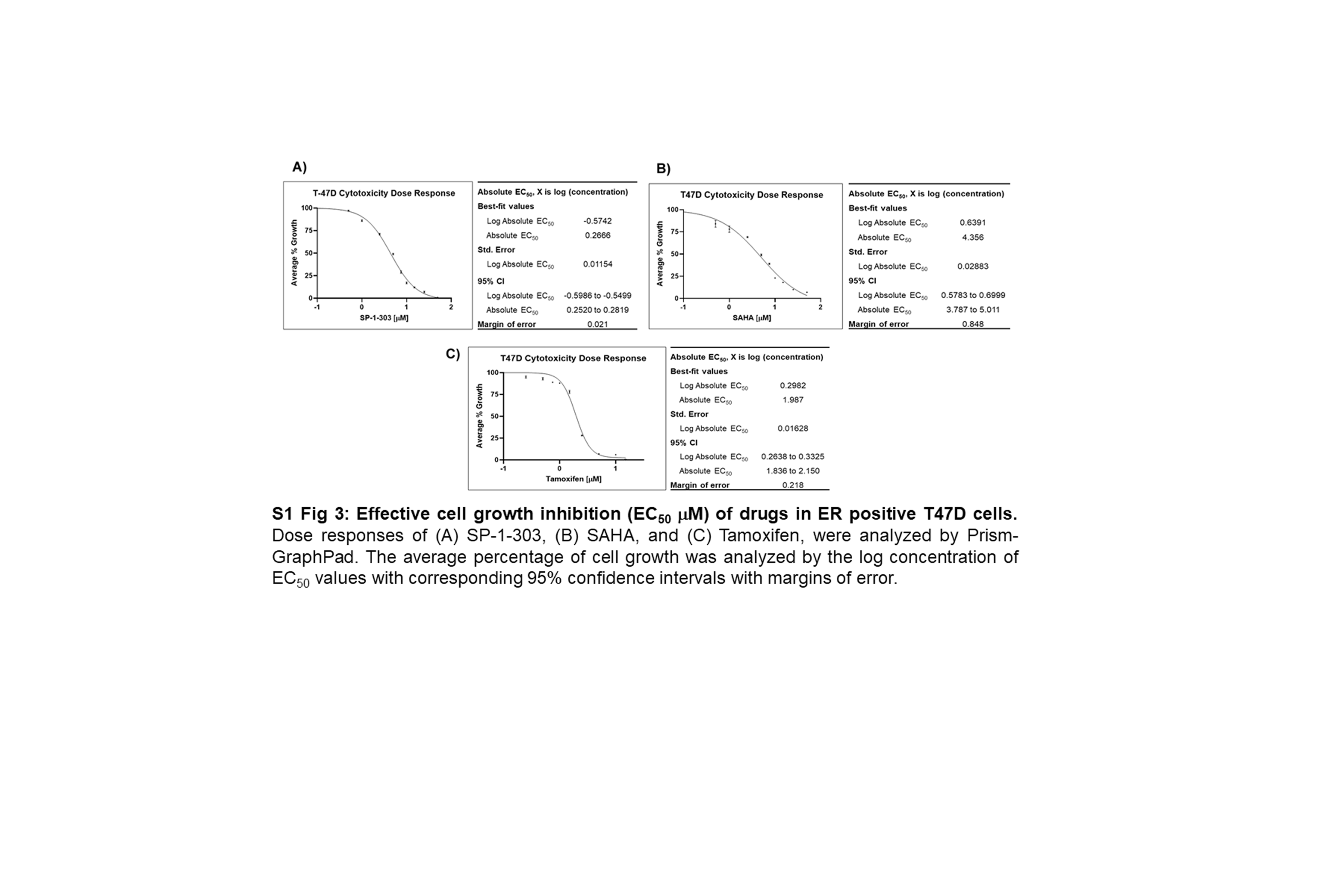

Supplement: S3 Fig — Dose responses of (A) SP-1-303, (B) SAHA, and (C) Tamoxifen, were analyzed by Prism-GraphPad. The average percentage of cell growth was analyzed by the log concentration of EC50 values with corresponding 95% confidence intervals with margins of error. (TIF) [file pone.0306168.s003.tif]

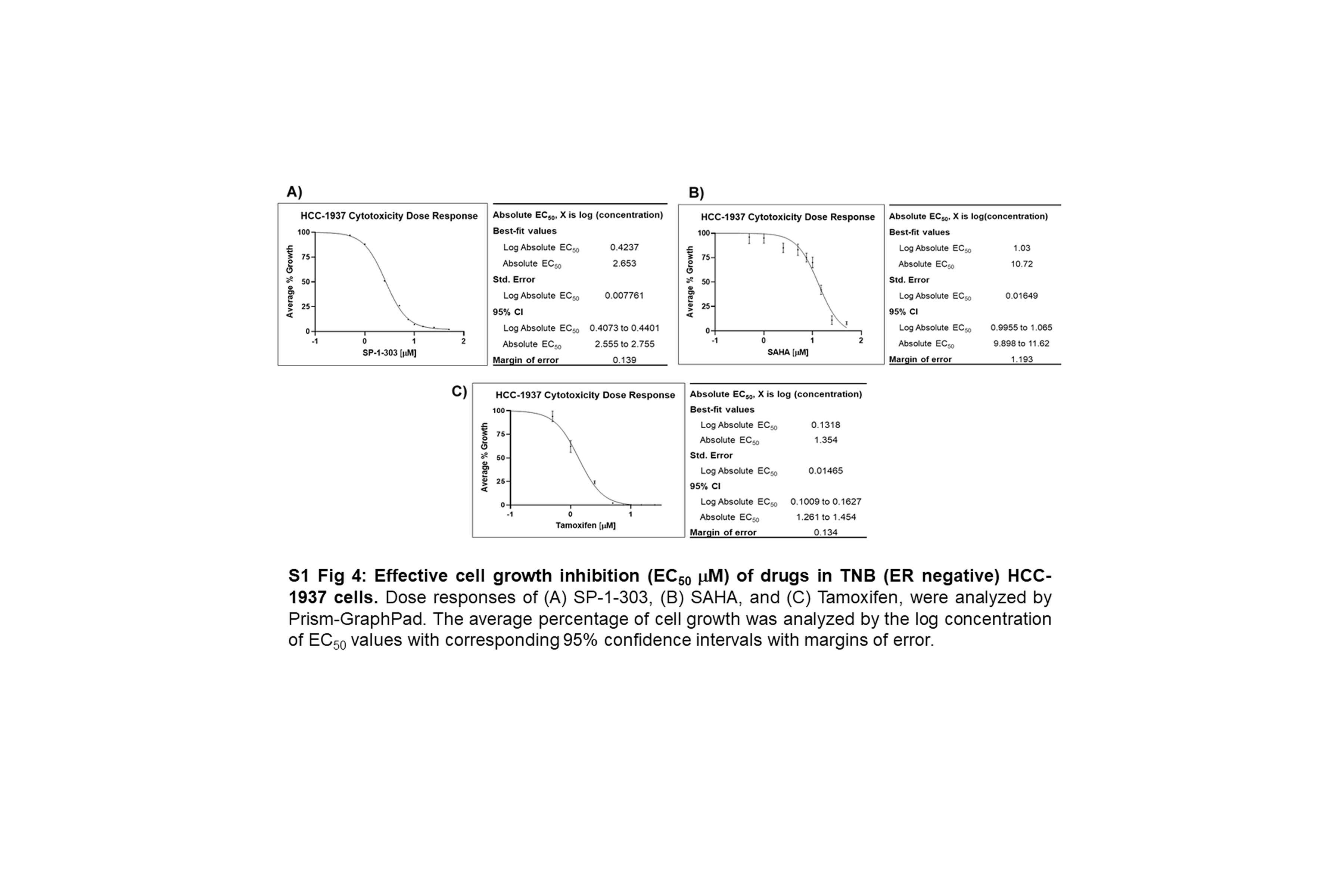

Supplement: S4 Fig — Dose responses of (A) SP-1-303, (B) SAHA, and (C) Tamoxifen, were analyzed by Prism-GraphPad. The average percentage of cell growth was analyzed by the log concentration of EC50 values with corresponding 95% confidence intervals with margins of error. (TIF) [file pone.0306168.s004.tif]

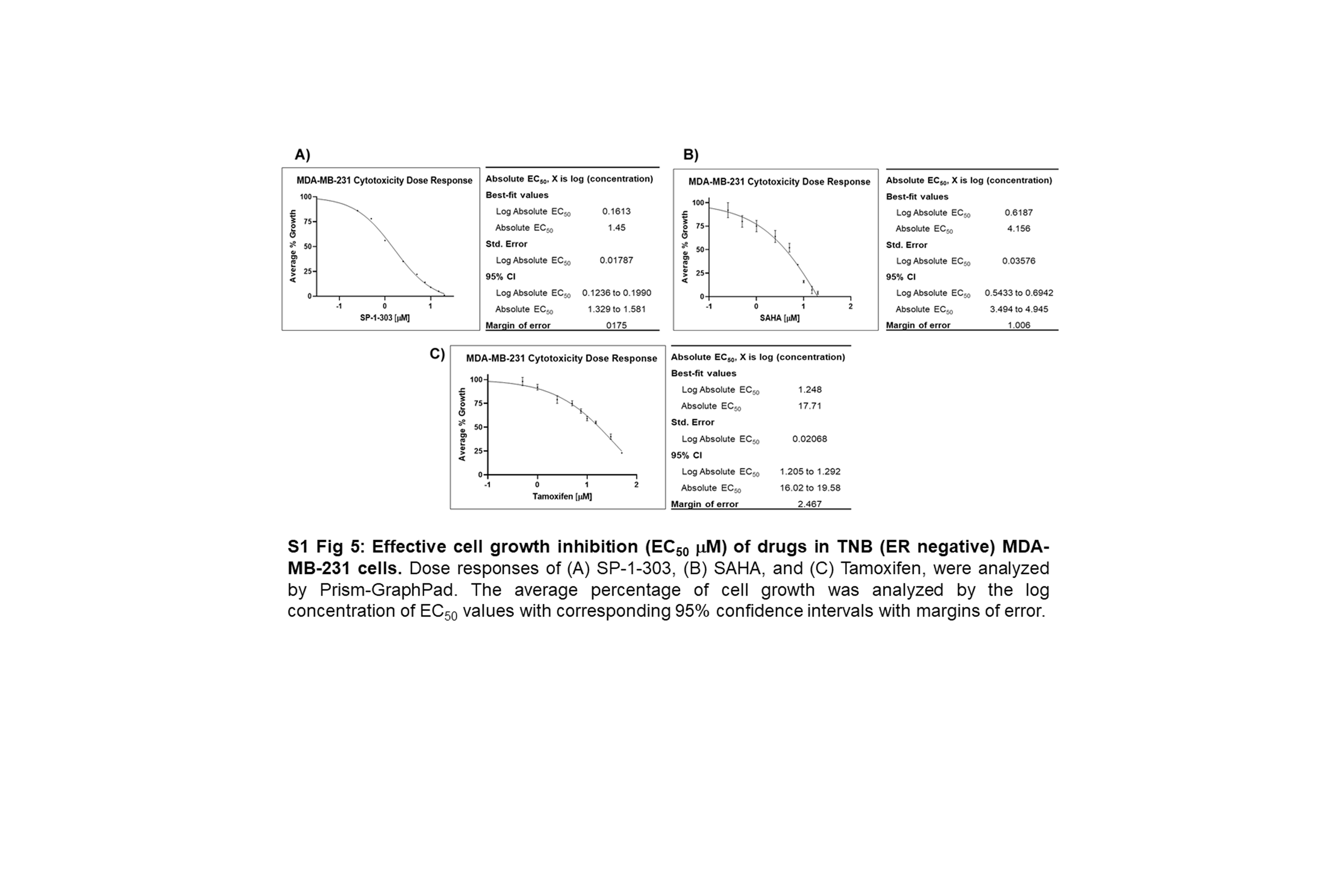

Supplement: S5 Fig — Dose responses of (A) SP-1-303, (B) SAHA, and (C) Tamoxifen, were analyzed by Prism-GraphPad. The average percentage of cell growth was analyzed by the log concentration of EC50 values with corresponding 95% confidence intervals with margins of error. (TIF) [file pone.0306168.s005.tif]
