# Supplementary material for: Dual-targeting class I HDAC inhibitor and ATM activator, SP-1-303, preferentially inhibits estrogen receptor positive breast cancer cell growth
Source: PLoS One. 2024 Jul 15;19(7):e0306168. doi: 10.1371/journal.pone.0306168 (PMC11249239; doi:10.1371/journal.pone.0306168)
Supplement: S1 Raw images — (PDF) [file pone.0306168.s006.pdf]

S1_raw_images_for Fig 2

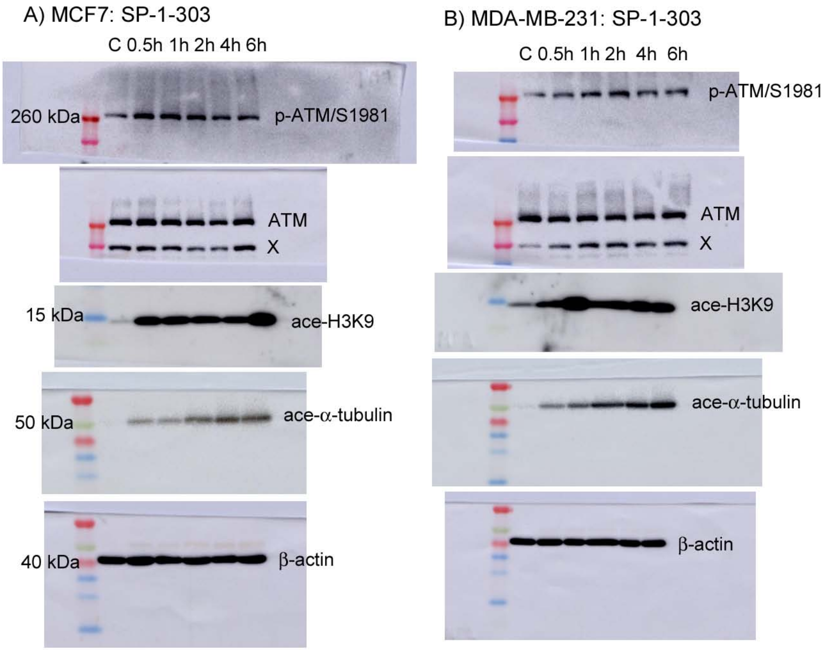

Images were captured by Amersham Imager 600.

S1_raw_images_for Fig 4A&B

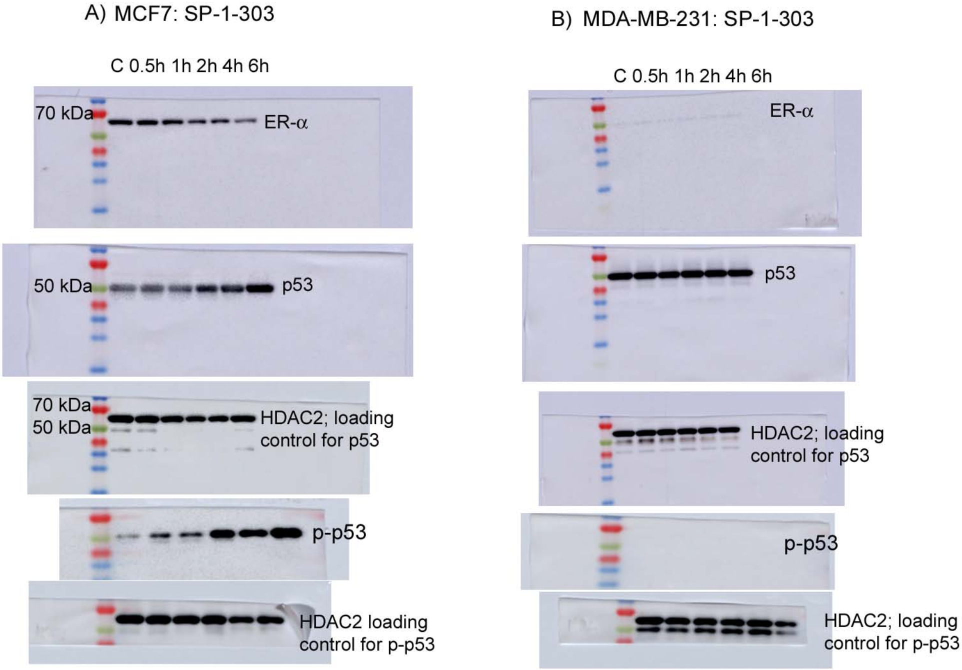

Images were captured by Amersham Imager 600.

S1_raw_images_for Fig 4C

C)

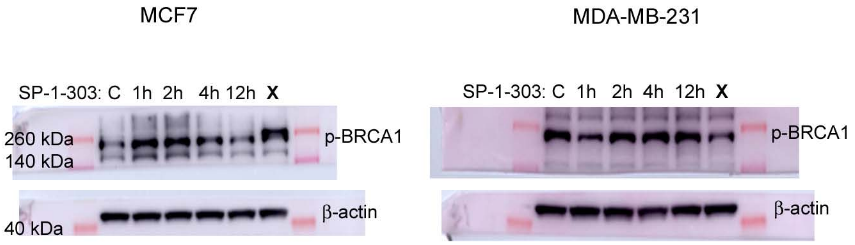

Images were captured by using Amersham Imager 600.
